# Assessment and topographic characterization of locoregional recurrences in head and neck tumours

**DOI:** 10.1186/s13014-015-0345-4

**Published:** 2015-02-15

**Authors:** Brigida Costa Ferreira, Rui Vale Marques, Leila Khouri, Tânia Santos, Pedro Sá-Couto, Maria do Carmo Lopes

**Affiliations:** I3N Physics Department, Aveiro University, Aveiro, Portugal; Radiation Therapy Department, Portuguese Oncology Institute of Coimbra Francisco Gentil (IPOCFG), Coimbra, Portugal; Physics Department, Coimbra University, Coimbra, Portugal; Center for Research and Development in Mathematics and Applications and Department of Mathematics, Aveiro University, Aveiro, Portugal; Medical Physics Department, Portuguese Oncology Institute of Coimbra Francisco Gentil, Coimbra, Portugal; Serviço de Física Médica, Inst. Port. Oncology of Coimbra, Av. Bissaya Barreto 3000-075, Coimbra, Portugal

**Keywords:** Head and neck tumours, Radiation therapy, Characterization of tumour recurrences, Geometric methods, Dosimetric assessment

## Abstract

**Purpose:**

To evaluate the differences between three methods of classification of recurrences in patients with head and neck tumours treated with Radiation Therapy (RT).

**Materials and methods:**

367 patients with head and neck tumours were included in the study. Tumour recurrences were delineated in the CT images taken during patient follow-up and deformable registration was used to transfer this volume into the planning CT. The methods used to classify recurrences were: *method CTV* quantified the intersection volume between the recurrence and the Clinical Target Volume (CTV); *method TV* quantified the intersection between the Treated Volume and the recurrence (for method CTV and TV, recurrences were classified in-field if more than 95% of their volume were inside the volume of interest, marginal if the intersection was between 20-95% and outfield otherwise); and *method COM* was based on the position of the Centre Of Mass of the recurrence. A dose assessment in the recurrence volume was also made.

**Results:**

The 2-year Kaplan-Meier locoregional recurrence incidence was 10%. Tumour recurrences occurred in 22 patients in a mean time of 16.5 ± 9.4 months resulting in 28 recurrence volumes. The percentage of in-field recurrences for methods CTV, TV and COM was 7%, 43% and 50%, respectively. Agreement between the three methods in characterizing individually in-field and marginal recurrences was found only in six cases. Methods CTV and COM agreed in 14. The percentage of outfield recurrences was 29% using all methods. For local recurrences (in-field or marginal to gross disease) the average difference between the prescribed dose and *D*_98%_ in the recurrence volume was -5.2 ± 3.5% (range: -10.1%-0.9%).

**Conclusions:**

The classification of in-field and marginal recurrences is very dependent on the method used to characterize recurrences. Using methods TV and COM the largest percentage of tumour recurrences occurred in-field in tissues irradiated with high doses.

**Electronic supplementary material:**

The online version of this article (doi:10.1186/s13014-015-0345-4) contains supplementary material, which is available to authorized users.

## Introduction

Intensity Modulated Radiation Therapy (IMRT) offers significant improvements in treatment quality for head and neck tumour cases especially in terms of toxicity [1-3]. The steep dose gradients obtained with IMRT dose distributions, compared with 3D conformal RT, allows better target volume coverage and normal tissue sparing. Accurate target volume delineation, combined with the determination of institutional margin criteria, becomes then crucial because uncertainties in structures definition combined with deviations in patient positioning relatively to the planned treatment may significantly affect outcome.

With the technological developments in RT, information about the patterns of failure of treatment techniques currently in practice are fundamental to identify the steps of the RT workflow where more aggressive strategies or practice improvements may bring additional therapeutic benefits. The steep penumbras of IMRT dose distributions, aimed at protecting radiosensitive normal tissues, raised some initial concerns about geographical misses in target borders [4,5]. However, in previous studies the majority of locoregional treatment failures originated in regions irradiated with high doses and seldom from the boundaries between the target volume and the parotids, oral cavity or submandibular glands [6-8].

The criteria used to judge the type of recurrence is fundamental. Recurrences classified as in-field may indicate the need for a dose escalation in the target volume. A high incidence of marginal recurrences may suggest the need to increase clinical and setup margins to account for uncertainties in delineation and patient positioning, respectively, provided that acceptable rates of complications are still obtained. Alternatively, more sophisticated strategies combining 3D Image Guided RT with Adaptive RT may have to be implemented on a larger scale [9]. A high rate of outfield recurrences would recommend a revision in target volume definitions or other treatment modalities.

The most common method of classification of locoregional recurrences is based on the quantification of the intersection between the volume of the recurrence and the 95% prescription isodose [5,6,8,10]. This isodose surface encompasses the treated volume or the tissues that received the therapeutic dose. Therefore this treated region is more relevant for the evaluation of failure patterns than delineated target volumes. Raktoe et al [7] quantified the volume of intersection of the recurrence volume with the initial target volume but proposed a new method to categorize recurrences. The centre of mass of the recurrence volume was used to define its origin assuming an isotropic tumour growth. The location of this centre of mass relatively to target margins defined the recurrence type.

In alternative to geometric evaluation methods, the analysis of the dose in the recurrence volume has also been made [5,8,11]. For local recurrences, the tissues where the recurrence later developed were irradiated with more than 85% of the prescription dose. For regional recurrences coverages of at least 52% of the prescribed dose were obtained [5,8].

Using the patterns of failure of patients with head and neck tumours treated with RT at the Portuguese Oncology Institute of Coimbra Francisco Gentil (IPOCFG), the purpose of this retrospective study was the characterization of locoregional recurrences using the geometric methods described above and to evaluate the disparity in the classification of recurrences when different methods are used. These results were also compared with a dosimetric analysis.

## Materials and methods

### Patients and treatments

367 patients with head and neck tumours, treated at IPOCFG from May 2007 to June 2013 were included in the study. Palliative and re-irradiated patients were excluded. Patient and disease characteristics are summarized in Table 1. Patient group was composed by 308 men and 59 women with a median age of 58.1 years (range: 12-88 years). Mean follow-up time was 16.6 ± 12.2 months (range: 40 days-6.1 years). Tumour cases were divided in larynx (22.9%), oral cavity (20.2%), oropharynx (19.1%), nasopharynx (12.3%), pharyngeal-laryngeal (10.4%), hypopharynx (7.4%) and others (7.9%). Tumour stage was divided in I-II (19.6%) and III-IV (80.4%). Definitive RT was offered to 58.9%.

**Table 1:** **Patient characteristics**

**Characteristic**	**N (%)**
Gender	
Male	308 (83.9)
Female	59 (16.1)
Site	
Larynx	84 (22.9)
Oral cavity	74 (20.2)
Oropharynx	70 (19.1)
Nasopharynx	45 (12.3)
Pharyngeal-laryngeal	38 (10.4)
Hypopharynx	27 (7.4)
Others	29 (7.9)
T stage	
1-2	188 (51.8)
3-4	175 (48.2)
N stage	
0-1	164 (45.2)
2-3	199 (54.8)
AJCC stage	
I	37 (10.2)
II	34 (9.4)
III	69 (19.1)
IV	222 (61.3)
Type treatment	
Post-operative	151 (41.1)
Definitive	216 (58.9)
RT Treatment technique	
3DCRT	57 (15.5)
dIMRT	98 (26.7)
rIMRT	155 (42.2)
IMRT	57 (15.5)

3DCRT stands for 3D Conformal RT, dIMRT stands for forward optimized IMRT (using 15-25 segments), rIMRT refers to inversely optimized IMRT planned with a small number of segments (30-55 segments) while IMRT was planned with about 70-80 segments.

Patients were treated in supine position immobilized with a thermoplastic mask. A Computer Tomography (CT) scan without contrast and with 1.5 or 3 mm slice thickness was acquired for treatment planning. For selected patients additional imaging, like positron emission tomography and/or magnetic resonance imaging, was performed for better target volume delineation. Target volume delineation followed Grégoire et al [12] recommendation for node negative neck and Grégoire et al [13] proposal for CTV in node-positive and post-operative neck. Prescription doses to primary tumour volume (CTV-T, post-operative or definitive) and large adenopathies (CTV-N) ranged from 59.4-70.2 Gy and to high and low risk lymph nodes (CTV-N1 and CTV-N2, respectively) ranged from 50.4-59.4 Gy. Treatment planning objectives followed ICRU 83 recommendations and planning evaluation followed RTOG H-0022 [14,15]. 95% of the volume of the PTVs should receive 95% of the prescribed dose and not surpass 107%. A maximum dose constraint in the spinal cord and brain stem of 45 Gy and 54 Gy, respectively, was imposed. As objective, among others, the mean dose in the parotids should be as low as possible aiming at achieving less than 26 Gy [14]. The main organs at risk included spinal cord, brainstem, parotid glands, thyroid, mandible and others considered relevant for each pathology.

Simpler target volumes were irradiated with 3D Conformal Radiation Therapy (3DCRT) where up to 10 beams were sufficient to reach treatment goals. These included 16% of the patients mostly with stage I-II tumours of the larynx and oral cavity. More complex cases were irradiated with IMRT. In 2006 forwardly optimized IMRT (direct, dIMRT) was implemented and used until 2012. This technique used 5-7 gantry directions with a total of 15-25 segments manually optimized. Dose fractionation schedule was based in the delivery of five fractions, of 1.8 Gy, per week delivered by two or three sequential plans. This technique was progressively replaced by inversely optimized step and shoot IMRT since 2008 using 5-9 6MV photon beams (Oncor Avant-Garde from Siemens) and 30-55 segments (rapid, rIMRT). Inversely optimized step and shoot IMRT, using 5-9 beams equidistant or angle optimized and an average of 70-80 segments (IMRT), was used when a smaller number of segments was not sufficient and patient could sustain longer irradiation times. These were mostly nasopharynx, and more complex oropharynx, hypopharynx and pharyngeal-laryngeal cases. With inverse IMRT, dose integration of at least two prescription dose levels was made [16].

The local protocol for Image Guided RT was followed for patient positioning verification and replanning needs. In summary, patient positioning was verified before treatment in the first three fractions and then weekly using two orthogonal 2D portal images for 3DCRT and dIMRT while MV-CBCT for inverse IMRT was used instead. A tolerance action level for the difference between the patient and the machine isocenter of 3 mm was adopted [17]. Furthermore, around the middle of RT or when significant anatomic variations were observed, a second CT was performed to evaluate differences in the dose distribution compared to the planned dose. For this patient cohort, Adaptive RT was performed in 22 patients.

NCCN guidelines were adopted for patient follow-up.

The cumulative incidence of local and locoregional recurrences were calculated using the Kaplan-Meier method counting the first RT fraction as the starting day.

### Classification of locoregional recurrences

Tumour recurrences were diagnosed in 22 patients and diagnostic images could be retrieved for 21. Recurrences were defined as the reappearance of the disease after complete tumour response to the initial treatment protocol, i.e., absence of visible disease during a minimum period of four months after the clinical evidence of complete tumour remission. Patients with residual or persistent disease after RT were not included in this study.

Tumour recurrences were delineated in the CT images taken within the clinical follow-up protocol. Deformable registration, using Velocity AI (version 2.8.1), was used to co-register this CT set with the planning CT using the local clinical protocol for image registration [18]. In summary, a region of interest around the recurrence volume in the follow-up CT was defined as the image volume of interest to be deformed. First, a rigid registration was made between the two sets of CT images followed by deformable registration using the pre-defined filters in Velocity AI. The recurrence structure was then transferred into the planning CT and clinical validation was made by the radiation oncologist. This deformed structure is mentioned, in here, as the recurrence volume albeit it refers to the tissues inside that region in the planning CT and not the recurrence itself. Geometric and dosimetric analysis was made assuming that the point of origin of the recurrence is an undetermined point within this deformed recurrence volume where at least one clonogenic cell survived radiation therapy. Repopulation of this surviving clonogenic cell or cells generated the macroscopic tumour volume, or recurrence, whose cells were in fact never irradiated. Although algorithms for elastic deformation still need to be biologically and physically validated, these were used in this study as the elastic deformation between two sets of images of the same patient provides today’s most accurate results.

Three methods were used to classify tumour recurrences. *Method CTV* quantified the intersection volume between the recurrence and the Clinical Target Volume (CTV). *Method TV* quantified the intersection between the Treated Volume (TV, defined as the 95% isodose [19]) and the recurrence volume. Both for method CTV and TV, recurrences were classified in-field if more than 95% of their volume were inside the volume of interest, marginal if the intersection was between 20-95% and outfield otherwise. *Method COM,* as proposed by Raktoe et al [7], was based on the position of the Centre Of Mass (COM) of the deformed recurrence volume (recurrence structure transferred into the planning CT), relatively to the nearest target volume border, i.e., for local recurrences the target volume corresponding to gross disease and for regional recurrences elective target volumes. With this method recurrences were defined as marginal when the minimum distance between the centre of mass and the closest target border was within ±3 mm. This tolerance accounts for uncertainties in delineation, empirically estimated to be between 2-3mm, and registration deformation, quantified to be between 3-5% or 1 mm [18]. Negative values indicate that the centre of mass was found outside the planning target volume and recurrence was defined as outfield while a positive value classifies the recurrence as in-field.

**Figure 1:**
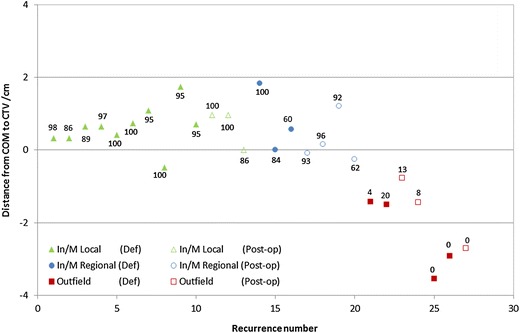
**Comparison between method COM and TV.** Symbols in the figure show the results for the COM method. The values around the symbols show the results of method TV (intersection volume in percentage). In/M Local refers to local recurrences that were classified either as in-field or marginal depending on the classification method. Closed symbols show cases treated with definitive RT while for cases with open symbols the treatment was post-operative. Recurrences were ordered by recurrence type, by definitive or post-operative, by prescribed dose and finally by recurrence volume.

**Figure 2:**
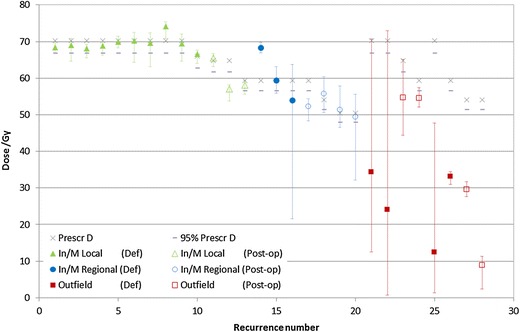
**Dosimetric analysis in the recurrence volume.** Dosimetric comparison between the dose in the deformed recurrence volume, the prescribed dose and 95% of the prescribed dose (shown by the X and -, respectively). The dose range in the recurrence volume is shown by the vertical bars (range *D*_98%_-*D*_2%_).

Throughout this study recurrences were divided into three main groups: local, regional and outfield. The motivation for this grouping was twofold. First, the probability of treatment failure near gross disease may be different than near microscopic disease. Therefore, there must be a clear separation between local recurrences (associated to gross disease, i.e., primary tumour and large adenopathies) from regional recurrences (associated to spread of microscopic disease, i.e., target volumes delineated to account for lymphatic spread). Outfield recurrences should theoretically have an origin outside delineated target volumes and therefore ideally should have no association with gross or known patterns of microscopic disease. Second, the classification of recurrences using different methods was consistent in the definition of outfield recurrences. However there was a large disparity in the classification of in-field and marginal recurrences which were clearly related to local and regional recurrences. Thus *local* in-field or marginal recurrences will be referred as local; while *regional* in-field or marginal as regional (triangles and circles, respectively, in Figure 1 and Figure 2). Remaining cases will be referred as *outfield* (squares in Figure 1 and Figure 2).

For the dosimetric analysis, the recurrence structure deformed into the planning CT was used to determine the mean dose, the maximum significant dose (*D*_2%_) and the minimum significant dose (*D*_98%_) [15] for the overall treatment delivered to each patient. All plans initially planned with pencil beam were re-calculated using collapsed-cone algorithm (six patients). Dose distributions have been corrected for incomplete treatment delivery (two cases).

## Results

The 24 months Kaplan-Meier estimate for local and locoregional recurrence was 7.4% and 10.3%, respectively (Additional file 1: Figure S1). Tumour recurrences were observed in 22 patients (Table 2). Mean time for locoregional failure was 16.5 ± 9.4 months (range: 6.8-35.0 months). Most recurrences were found in patients with high stage tumours: III-IV (81.8%). Most common primary tumour site for recurrence events was: hypopharynx 5/27 (18.5%), oropharynx 5/70 (7.1%), oral cavity 5/74, larynx 4/84 (4.8%), nasopharynx 2/45 and pharyngeal-laryngeal 1/45. Recurrences were identified in 9/57 (9.2%) patients irradiated with dIMRT, in 5/57 (8.8%) with 3DRCT, in 6 (3.9%) with rIMRT and in 2 (3.5%) irradiated with IMRT (Table 2). Mean time for recurrence diagnosis was: for dIMRT 20.8 ± 9.8 months, for 3DCRT 9.2 ± 2.4 months and for rIMRT 13.2 ± 5.2 months.

**Table 2:** **Patient and treatment characteristics for the patients with recurrence**

**Primary tumour site**	**Stage**	**Type RT**	**Type CH**	**Dpresc/Gy/#fx**	**Ddeliv CTV-T/Gy**	**RT tech**	**Recurrence location**	**Diagn./months**	**Rec vol./cm3**
Oral cavity (tongue)	III	Adjuvant (Post-op)		59.1/30	59.0	3DCRT	Mobile Tongue + Cervical	6.7	2.0 + 27.6
Oral cavity (mand. gingivae)	IVA	Adjuvant (Post-op)		59.4/31	58.7	dIMRT	Mandibular gingivae	7.8	7.0
Oral cavity (tongue)	IVA	Adjuvant (Post-op)		59.0/31	59.8	dIMRT	Cervical	9.0	27.1
Oral cavity (hard palate)	IVA	Concomitant	Cisplatin	70.2/39	70.2	3DCRT	Pharynx + Cervical	8.5	16.6 + 82.2
Oral cavity (max. gingivae)	IVA	Concomitant (Post-op)	Cisplatin	64.8/36	65.3	rIMRT	Maxillary gingivae	8.7	9.9
Oropharynx (tonsil)	II	Intensive		70.2/33	69.8	rIMRT	Tonsil	22.8	clinical
Oropharynx (tonsil)	IVA	Adjuvant (Post-op)		59.4/33	57.0 (pb)	dIMRT	Base tongue	21.1	4.0
Oropharynx (base tongue)	IVA	Sequential	TPF	69.3/33	69.2	rIMRT	Base tongue	9.8	36.6
Oropharynx (base tongue)	IVA	Sequential	TPF	70.2/39	68.1 (pb)	dIMRT	Cervical	31.2	17.1
Oropharynx (base tongue)	IVA	Concomitant	Cisplatin	69.6/33	68.7	IMRT	Tonsil + Cervical + Submandid.	7.3	0.4 + 0.5 + 2.7
Hypopharynx (pyriform sinus)	IVA	Concomitant (Post-op)	Cisplatin	64.8/33	56.9/29*	rIMRT	Posterior wall + Cervical	9.8	10.3 + 24.1
Hypopharynx (posterior wall)	IVA	Intensive		69.0/33	68.0	rIMRT	Cervical	15.4	0.6 + 1.0
Hypopharynx (pyriform sinus)	IVA	Sequential	PF	70.2/39	67.7 (pb)	dIMRT	Cervical	26.7	0.3
Hypopharynx (pyriform sinus)	IVA	Sequential	PF	70.2/39	70.1	rIMRT	Pyriform sinus	9.6	8.0
Hypopharynx (pyriform sinus)	IVB	Sequential	TPF	70.2/39	72.4 (pb)	dIMRT	Cervical	23.7	15.2
Larynx (glottis)	I	Intensive		66/33	65.9	3DCRT	Larynx	12.4	14.8
Larynx (glottis)	II	Intensive		70.0/35	69.9	3DCRT	Larynx	9.2	19.4
Larynx (supraglottis)	IVA	Concomitant	Cisplatin	70.2/39	69.4 (pb)	dIMRT	Larynx	32.6	1.3
Larynx (supraglottis)	IVA	Concomitant (Post-op)	Cisplatin	64.8/36	62.6/35*	dIMRT	Glottis	8.8	1.7
Nasopharynx	IIB	Intensive		70.2/39	69.4	rIMRT	Cervical	16.0	2.7
Nasopharynx	IVB	Concomitant	Cisplatin	70.2/33	75.9 (pb)	IMRT	Right orbit	34.4	11.9
Pharyngeal-laryngeal	IVA	Adjuvant (Post-op)		59.4/33	59.7	dIMRT	Peritracheostomy + Cervical	25.5	0.5 + 44.0

Dpresc and Ddeliv is the prescribed dose and the mean dose delivered to the primary tumour corrected for incomplete treatment delivery and recalculated with the collapsed-cone dose algorithm.

*Failure to complete prescribed treatment, (pb) refers to the dose calculation algorithm used for treatment planning.

Recurrences were classified as local to gross disease in 10 cases (closed triangles in Figure 1), local to the tumour bed in three cases (open triangles) and regional in seven cases (circles). Eight recurrences were classified as outfield (squares). Only one recurrence occurred in the vicinity of the parotid.

Consistency between the three methods in characterizing in-field and marginal recurrences was found in two and four cases, respectively. Comparing method TV and COM, consistency in classifying in-field and marginal recurrences was found in 14 cases (values and symbols in Figure 1, respectively). All methods were consistent in the classification of the eight outfield recurrences.

Using method CTV, one and twelve recurrences were considered in-field and marginal, respectively, to local disease; and one and six were considered in-field and marginal to regional target volumes, respectively (Table 3).

**Table 3:** **Classification of 20 out of the 28 recurrences volumes using method CTV, TV and COM**

	**Method**	**In-field**	**Marginal**	**Outfield**	**N**
**CTV-T (primary tumour)**	CTV	1	9	0	10
	TV	8	2	0	
	COM	8	1	1	
**CTV-N (large adenopathies)**	CTV	0	3	0	3
	TV	2	1	0	
	COM	3	0	0	
**CTV-N1 (high risk lymph nodes)**	CTV	1	4	0	5
	TV	2	3	0	
	COM	2	3	0	
**CTV-N2 (low risk lymph nodes)**	CTV	0	2	0	2
	TV	0	2	0	
	COM	1	1	0	

The remaining eight recurrences were classified as outfield by all evaluation methods.

N is the number of cases in each category.

With method TV, ten and three were classified in-field and marginal, respectively, to the highest dose level (prescribed to gross disease). Two and five were considered in-field and marginal, respectively, to lowest dose levels prescribed to the lymph nodes (intersection volumes in Figure 1).

In method COM, eleven, one and one were classified as in-field, marginal and outfield to local disease, respectively; three and four were classified as in-field and marginal to regional volumes, respectively.

The symbols in Figure 1 show the distance from the centre of mass of the recurrence volume to the nearest CTV margin. The average distance, between the centre of mass of the recurrence and the nearest target margin, for local recurrences was 7.1 ± 4.5 mm (range: 0.0-17.4 mm) and for regional recurrences was 4.9 ± 7.7 mm (range: -2.6-18.3 mm). The average distance for the first four outfield recurrences (#21-24) was -1.3 ± 0.3 cm (range: -1.5- -0.8 cm) and for the outfield recurrences #25-28 was -4.1 ± 2.2 cm (range: -7.4- -2.7 cm).

The average mean dose and average *D*_98%_ in the recurrence volumes for each group are shown in Table 4. For local recurrences (triangles in Figure 2), the average difference between the prescribed dose and the mean dose in the recurrence was -0.5 ± 2.3% (range: -3.0-5.7%), the average difference between the prescribed dose and *D*_98%_ was -5.2 ± 3.5% (range: -10.1-0.9%) and the average difference between *D*_95%_ in the initial target volume and *D*_98%_ in the recurrence was -0.2 ± 3.7% (range: -5.4-6.2%). For regional recurrences (circles in Figure 2), the average difference between the prescribed dose and the mean dose in the recurrence was 1.4 ± 7.9% (range: -9.3-15.0%) and the average difference between the prescribed dose and *D*_98%_ in the recurrence was -17.9 ± 27.4% (range: -63.8-12.6%).

**Table 4:** **Summary of the dose assessment for the recurrence volumes**

**Type**	**Group**	**Vol ± SD/cm** ^3^	**Presc D/Gy**	***D***_mean_ **± SD [min-max]**	***D***_98%_ **± SD [min-max]**
**Local**	**CTV-T (def.)**	14.0 ± 14.4	70.2	69.6 ± 2.4 [66.6 – 74.2]	65.5 ± 2.5 [63.1 – 70.8]
	**CTV-T (post-op)**	9.3 ± 12.8	59.4-64.8	60.3 ± 4.6 [57.1 – 65.6]	58.4 ± 5.8 [54.8 – 65.1]
	**CTV-N (3 cases)**	1.4 ± 1.1	70.2	69.1 ± 0.8 [68.4 – 69.9]	66.6 ± 1.8 [64.7 – 68.3]
**Regional**	**CTV-N1**	14.5 ± 18.0	54-59.4	57.9 ± 6.4 [52.2 – 68.3]	48.6 ± 16.8 [21.5 – 66.9]
	**CTV-N2**	15.6 ± 16.3	50.4	50.4 ± 1.3 [49.4 – 51.3]	39.3 ± 10.2 [32.1 – 46.5]
	**Outfield**	19.5 ± 26.8		31.4 ± 17.0 [8.9 – 54.6]	21.5 ± 20.3 [0.7 – 52.1]

Vol refers to the average volume of the recurrences for each group.

CTV-T stands for the clinical target volume of the primary tumour, CTV-N for large adenopathies, CTV-N1 for high risk lymph nodes and CTV-N2 for low risk lymph nodes. Def stands for definitive RT and post-op to post-operative.

## Discussion

In this retrospective study the locoregional patterns of failure for head and neck cancer patients irradiated at IPOCFG were reported and the topographic characterization of recurrences analysed. At 24 months follow-up time, the probability of locoregional recurrences was 10%. Patients with hypopharynx tumours had the worst disease outcome with a recurrences incidence of 19% compared to 7% for patients with tumours in oropharynx and oral cavity or 5% for larynx and nasopharynx tumour cases. Although survival statistics are consistent with others [3,5], these should be regarded with caution due to the short mean follow-up time and the heterogeneity of the population varying in terms of tumour type, stage, treatment protocol, RT technique and fractionation. A deeper understanding of the causes for treatment failure for each tumour type and treatment technique is needed. However, the number of recurrences was too small to allow taking any further conclusions. Given the low incidence of locoregional recurrences, the identification of the localization of radioresistant malignant cells may be the best therapeutic approach aiming to improve tumour control without increasing the probability of complications to all patients.

Studies are consistent in pointing out the advantages of IMRT in the treatment of head and neck cancer [2,14]. Some initial concerns were highlighted about the consequence that the IMRT steep dose gradients could have in terms of the probability of tumour control [4,5]. In this study only one periparotid recurrence was observed. For that patient, given the proximity of a large adenopathy to the right parotid, priority was given to target volume coverage. No attempt was made to spare that structure that was irradiated with more than 56 Gy. At the time of the last follow-up appointment, 43 months after RT, that patient had good response to stimulation and was thus graded with G1 xerostomia. For this population cohort a fair comparison in terms of treatment technique could not be made due to a bias in patient selection. The RT technique used for the treatment of each patient is a consequence of both the evolution of techniques at IPOCFG and the therapeutic needs of each particular case. Although the treatment techniques with highest incidence of recurrences were dIMRT and 3DCRT, this was the group of patients with the longest follow-up time. Still for this very small sample of patients, the mean time for recurrence for dIMRT and rIMRT was 21 months and 13 months, respectively. A longer follow-up time is needed to validate these findings.

Three methods were employed to categorize recurrences in RT of head and neck tumour cases. All geometric methods were consistent in the classification of around 29% of outfield recurrences. However, for in-field and marginal recurrences, the classification of each recurrence was highly dependent on the method of evaluation used (Table 3). The percentage of in-field recurrences using method CTV, TV and COM was 7%, 43% and 50%, respectively. Even that the methods do not concur in their absolute evaluation, in relative terms method TV and COM categorized most recurrences as occurring in-field, as reported by others [6-8]. In-field recurrences may be a consequence of residual disease advocating for the need of a dose escalation. Despite the short follow-up time, a low rate of locoregional recurrences was achieved and an escalation in prescription dose will simultaneously raise the probability of complications for all patients. Therefore, radioresistant tumours should be identified through imaging and biomolecular and genetic assays in order to individualize and escalate prescription doses for more difficult cases. Better target volume delineation, based on different imaging modalities, would also help reducing marginal recurrences and help to identify distant ill nodes that may result in outfield recurrences.

Method TV is more relevant than method CTV because the evaluation is based on the volume that received a high dose thus making the analysis more insensitive to target volume delineation. Given today’s uncertainties associated with target volume definition, methods of recurrence classification independent from target volume delineation are preferable. The larger size of the treated volume, as defined by the 95% prescription isodose, compared with the volume of the CTV led to the reclassification of most recurrences from marginal with method CTV into in-field with method TV. The discrepancy in the results obtained between the two methods is highly dependent on the non-biologically based threshold value of the intersection volume used to separate in-field from marginal and marginal from outfield recurrences, generally 95% and 20%, respectively [10]. For the method CTV the upper threshold is critical for the number of recurrences that would be classified as in-field while for method TV this percentage would only slightly vary (Figure 1).

In this study outfield recurrences may have two different causes of treatment failure. Planned dosimetry for the patient cases that resulted in the first four outfield recurrences (#21-24 Figure 2) showed that target volume coverage, or irradiation technique, were adequate, i.e., more than 98% of the PTV was irradiated with 95% of the dose (except for one case where D98% was 93% of the prescribed dose aiming to spare the ipsilateral optical nerve). Most probable causes for treatment failure in these four cases may then have been: small clinical margins, uncertainties in patient positioning or treatment interruption (two patients did interrupt radiation therapy two and five days). These patients would have then benefited from wider clinical or setup margins in the spot where the recurrence developed. According to the theoretic definition of less than 20% of intersection, these four recurrences were classified as outfield when conceptually they should have been more appropriately classified as marginal. The second group of outfield recurrences represents in fact true outfield events (#25-28 Figure 1 and Figure 2). Volumetric methods for recurrence classification are thus strongly dependent on the recurrence volume upon diagnosis and pathways of tumour growth and expansion.

The ideal method of classification of tumour recurrences would be based on the precise identification of recurrence origin. Geometric characterization of the recurrences using method COM assumes that the centre of mass of the recurrence volume represents its origin [7]. This method is a valid alternative to categorize recurrences, but it is only independent of the time of diagnosis if tumour growth is in fact isotropic. Otherwise, as for other methods, it depends as well on tumour size and the patterns of spread of malignant cells. Using method COM, 14/28 recurrences were classified as in-field as the distance between the centre of mass and the target had positive values above the uncertainty associated to target volume delineation and image registration (Figure 1). Five recurrences were classified as marginal as this distance was within ±3 mm [18]. This value was slightly smaller than the one estimated by Raktoe et al [7] because their analysis was based on rigid image registration. For the first four outfield recurrences (#21-24), the maximum distance between the centre of mass and target borders was -1.5 cm (Figure 1) and for the true outfield recurrences (#25-28) maximum distance to cervical targets was -7.6 cm. This was the case of a patient with a tumour in the oral cavity prescribed with unilateral irradiation that relapsed in the contralateral cervical lymph nodes (rec #28). For the distances reported, the first group of outfield recurrences was probably caused by inaccuracies in target volume delineation. Broader margins applied to all patients would result in an increase in the overall rate of complications. Therefore, the quality of radiation therapy may be improved by better target volume delineation by refining the usage of different imaging modalities. As well, the starting points of the second group of outfield recurrences most probably could only have been detected through additional imaging.

Deformable co-registration between the planning CT and the images acquired to diagnose the recurrence allowed to assess the dose delivered to the tissues included in the recurrence volume. For local recurrences, the dosimetric analysis indicates that in at least one point within the region where the recurrence later developed the dose was insufficient to eradicate all tumour cells. Comparing the dose in the recurrence volume with the prescribed dose: the mean dose in the recurrence volume was at least 97% of the prescribed dose, the minimum significant dose was larger than 90% of the prescribed dose and at least 95% of *D*_95%_ (triangles in Figure 2 and Table 4). As in any other dose statistics based on DVH, some care is recommended in the interpretation of these values as in that case the 3D spatial information is lost. A minimum dose in the recurrence volume of 90% of the prescribed dose may seem too low compared to ICRU recommendations, but corresponds to the worst case obtained. Furthermore, for all recurrences the minimum dose in its volume was outside the initial target volume and distant from its borders. Therefore, this difference may represent an underestimation of the dose delivered at the recurrence origin. If these recurrences are in fact a result of remaining disease, the small deviation between the minimum dose in local recurrences and the prescribed dose provides evidences about the radioresistance of head and neck tumour cases. A similar analysis for regional recurrences is not that straightforward because the dose range in this volume increases significantly as recurrences grows outside the target volume (Figure 2).

## Conclusions

For RT of head and neck cancers at our institution a low rate of locoregional recurrences was found. The classification of in-field and marginal recurrences is very dependent on the geometric method used for analysis because those rely on the recurrence volume upon diagnosis and pathways of tumour growth. Nevertheless with method TV and method COM the same general conclusions were taken in categorizing most of the recurrences as in-field. The dosimetric analysis supports the need to identify patients with radioresistant tumours aiming to individualize dose prescription.

## Additional file

Additional file 1: Figure S1. Kaplan-Meier estimates for the cumulative incidence of locoregional recurrence.
