# Supplementary figures and images for: Assessment and topographic characterization of locoregional recurrences in head and neck tumours
Source: Radiat Oncol. 2015 Feb 15;10:41. doi: 10.1186/s13014-015-0345-4 (PMC4619282; doi:10.1186/s13014-015-0345-4)

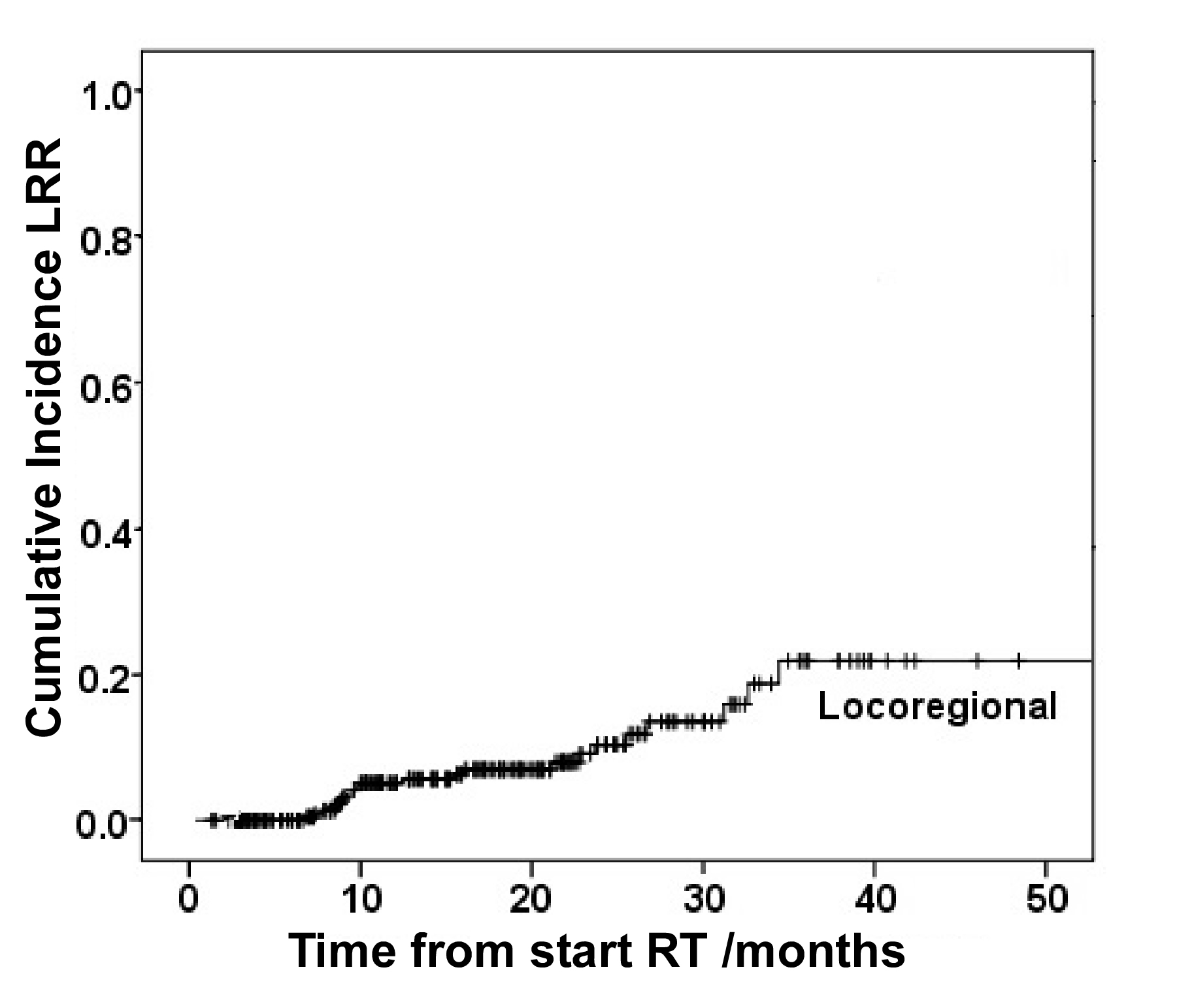

Supplement: Additional file 1: Figure S1. — Kaplan-Meier estimates for the cumulative incidence of locoregional recurrence. [file 13014_2015_345_MOESM1_ESM.jpeg]
